# Physiotherapists’ Use of Web-Based Information Resources to Fulfill Their Information Needs During a Theoretical Examination: Randomized Crossover Trial

**DOI:** 10.2196/19747

**Published:** 2020-12-17

**Authors:** Cailbhe Doherty, Arash Joorabchi, Peter Megyesi, Aileen Flynn, Brian Caulfield

**Affiliations:** 1 School of Public Health, Physiotherapy and Sports Science University College Dublin Dublin Ireland; 2 Insight Centre for Data Analytics O'Brien Centre for Science University College Dublin Dublin Ireland; 3 Department of Electronic & Computer Engineering University of Limerick Limerick Ireland; 4 Beacon Hospital Dublin Ireland

**Keywords:** evidence-based medicine, knowledge discovery, information seeking behavior, information dissemination, information literacy, online systems, point-of-care systems, mobile phone

## Abstract

**Background:**

The widespread availability of internet-connected smart devices in the health care setting has the potential to improve the delivery of research evidence to the care pathway and fulfill health care professionals’ information needs.

**Objective:**

This study aims to evaluate the frequency with which physiotherapists experience information needs, the capacity of digital information resources to fulfill these needs, and the specific types of resources they use to do so.

**Methods:**

A total of 38 participants (all practicing physiotherapists; 19 females, 19 males) were randomly assigned to complete three 20-question multiple-choice questionnaire (MCQ) examinations under 3 conditions in a randomized crossover study design: assisted by a web browser, assisted by a federated search portal system, and unassisted. MCQ scores, times, and frequencies of information needs were recorded for overall examination-level and individual question-level analyses. Generalized estimating equations were used to assess differences between conditions for the primary outcomes. A log file analysis was conducted to evaluate participants’ web search and retrieval behaviors.

**Results:**

Participants experienced an information need in 55.59% (845/1520) MCQs (assisted conditions only) and exhibited a mean improvement of 10% and 16% in overall examination scores for the federated search and web browser conditions, respectively, compared with the unassisted condition (*P*<.001). In the web browser condition, Google was the most popular resource and the only search engine used, accounting for 1273 (64%) of hits, followed by PubMed (195 hits; 10% of total). In the federated search condition, Wikipedia and PubMed were the most popular resources with 1518 (46% of total) and 1273 (39% of total) hits, respectively.

**Conclusions:**

In agreement with the findings of previous research studies among medical physicians, the results of this study demonstrate that physiotherapists frequently experience information needs. This study provides new insights into the preferred digital information resources used by physiotherapists to fulfill these needs. Future research should clarify the implications of physiotherapists’ apparent high reliance on Google, whether these results reflect the authentic clinical environment, and whether fulfilling clinical information needs alters practice behaviors or improves patient outcomes.

## Introduction

### Health Care Professionals’ Information Seeking Behaviors: A Brief History

The ubiquity of internet-connected smart devices in the clinical setting [1] and the availability of preappraised point-of-care evidence summaries [2,3] have changed how health care professionals seek, consume, and implement information in the care pathway [4]. For instance, a generation now separates the sample of health care professionals who participated in the seminal work of Covell et al [5] in 1985 and their digitally native descendants. That sample (47 medical physicians) reported having 2 clinical questions for every 3 patient visits and preferred textbooks, journals, and drug information indexes to fulfill their information needs [5]. Importantly, these findings are now irreconcilable with modern clinical archetypes as “computered sources were reported to be used least often*”* [5].

Since then, the delivery of clinically relevant information to the care pathway has progressed to smart devices capable of running stand-alone software apps. At present, an increasing proportion of health care professionals use these devices to inform their practice [1]. This change has been charted in thousands of studies [6] and syntheses of studies [7,8], which have demonstrated the use of web-connected smart devices for fulfilling clinical information needs [5,8-11]

### Empirical Research of Health Care Professionals’ Information Seeking Behaviors

Unfortunately, the current understanding of information-seeking and utilization behaviors is shaped by suboptimal empirical models. Self-report questionnaires [5,9], interviews [12], and think-aloud [13-15] methodologies may offer a feasible way to estimate how health care professionals perceive their own information-seeking behaviors, their frequency, the topics to which they relate, the resources they use to fulfill these needs, and the strategies they employ to harvest information from these resources. However, these behaviors can now be more directly observed using web server log file analysis [16-18].

To date, researchers have leveraged web logs to evaluate usage patterns for specific websites such as Wikipedia [19,20], PubMed [21,22], UpToDate [23], and web-based institutional health science libraries [16,17]. Typically reported usage metadata include the number of time- or user-dependent hits or sessions on the website [24,25], the most frequently searched topics [18,22], and the number of click-throughs from one section of the website to another [18].

Importantly, these analyses tend to be constrained to individual websites [22] or institutional portals [23], which redirect to a limited group of external websites. These constraints undermine the validity of modern information-seeking paradigms and bely our understanding of the specific digital information resources that are used in an unconstrained web environment to fulfil information needs. Health care professionals in the real world have unbounded web access; this does not reflect the sandboxes of preselected information resources that are evaluated in the available body of research. Furthermore, little work in the web log analysis literature has focused on how these resources can be used to answer clinical questions.

### Aims and Objectives of the Study

Therefore, the aim of this study is to evaluate the use of web-based resources for fulfilling clinical information needs. Specifically, our objectives were to conduct a randomized crossover trial, whereby a group of physiotherapists were subjected to a multiple-choice questionnaire (MCQ) examination, which they completed under 3 conditions: (1) assisted via a web browser with unconstrained web access, (2) assisted via a federated search engine “portal” app, and (3) unassisted.

By including both a federated search system and unconstrained web use in a single study design, we sought to evaluate the potentially mediating effects of the access tool on the kinds of resources being used and the time spent doing so. This study aims to address both hypothesis-confirming and hypothesis-generating research questions.

### Experimental Hypotheses

The confirmatory hypotheses were as follows:

1. Physiotherapists frequently encounter information needs when presented with simulated clinical questions [5,9-11].
2. Digital information resources available on the web can be used to fulfill these needs, resulting in a higher MCQ examination score in the assisted conditions [26].

The hypotheses that this research may generate relate to the following:

1. The specific web-based resources used by physiotherapists to fulfill their information needs.
2. The rate of answering correctly in the presence or absence of an information need and its relationship with the use or nonuse of a digital information resource.
3. Whether constraining physiotherapists to a limited number of web-based digital information resources (as in the federated search condition) is associated with a difference in the rate with which questions are answered correctly or the time spent in searching for information.

## Methods

### Ethics

Ethical approval for this randomized crossover trial was granted by the affiliate review board of the institution at which the authors were based (reference: LS-18-25). Study design, conduct, analysis, and results are reported according to the CONSORT-EHEALTH (Consolidated Standards of Reporting Trials of Electronic and Mobile Health Applications and Online Telehealth) checklist.

### Recruitment

Prospective participants included practicing physiotherapists who were recruited with the help of collaborating health care institutions. The study was advertised on the websites of these institutions, and a notice was sent throughout the recruitment period to prospective participants via the “staff bulletin” of the respective institutions. Physiotherapists were chosen for evaluation, as their information behavior has not been well addressed in the research literature to date, despite their prominent role in health care [27].

All individuals met the following inclusion criteria: adults (>18 years of age), currently employed by collaborating health-related institutions and involved with the management and treatment of patients, provided informed consent (which was obtained digitally during authentication with the federated search app).

### Protocol

Information needs were induced via the administration of an MCQ examination completed at 3 time points under 3 conditions in random order: (1) assisted by a standard web browser, (2) assisted by a federated search engine, and (3) unassisted.

One investigator (CD) organized an appropriate time and location to conduct all test sessions; to maximize participant retention, testing was conducted at a time and place that suited participants. Short-term rescheduling (ie, within 1 week of the original designated test time) was facilitated in the event of unexpected delays or schedule conflicts.

For the purpose of this study, we defined an information need in the assisted conditions as any instance in which a participant used an assistive technology to access a digital information resource to inform their choice of answer in the MCQ. In the “assisted by a web browser” condition, participants were provided with a web-connected laptop preinstalled with a web browser (Google Chrome). For the “assisted by a federated search engine” condition, participants were required to install and register an account with a free experimental federated search engine called *SciScanner* [28] on their smartphone.

#### The Multiple-Choice Questionnaire

Each theoretical examination comprised a 20-item MCQ with one best answer, derived from the Physiotherapy Competency Exam (PCE) [29] prepared by the Canadian Alliance of Physiotherapy Regulators and the United States National Physical Therapy Exam (NPTE) [30]. A bank of >1000 questions was compiled for each of the PCE and NPTE examinations [31,32]. During each examination, a different random subset was taken from this question bank for each participant for each of the 3 examinations they completed. The MCQ was administered on a tablet device using a commercially available testing software that administered random subsets of topic-tagged questions. Participants were assigned questions based on their chosen area of practice using these tags. The software also documented the question that was administered, its associated response, whether that response was correct or incorrect, and the time spent answering it [33]. Participants were informed that the examination was negatively marked to discourage guessing; however, they were not informed about the penalty for an incorrect answer. Participants were also informed that time was not to be considered a metric for performance, whereby they would not be penalized for finishing the examination in a longer period. In the “assisted” experimental conditions, participants were informed that they could opt to use their assigned system but did not have to if they did not deem it necessary or if they were sufficiently confident of their answer. In either case, participants were informed that they would not be penalized for using or not using assistance if it was available to them. The examination was proctored by the first author, who manually documented the frequency of information needs (defined by instances where participants chose to use either the standard web browser or the federated search engine for a given examination question).

#### Web and Federated Search Logs

Participants’ web logs and federated search logs were captured during the 2 assisted conditions.

##### Federated Search Logs

**Figure 1:**
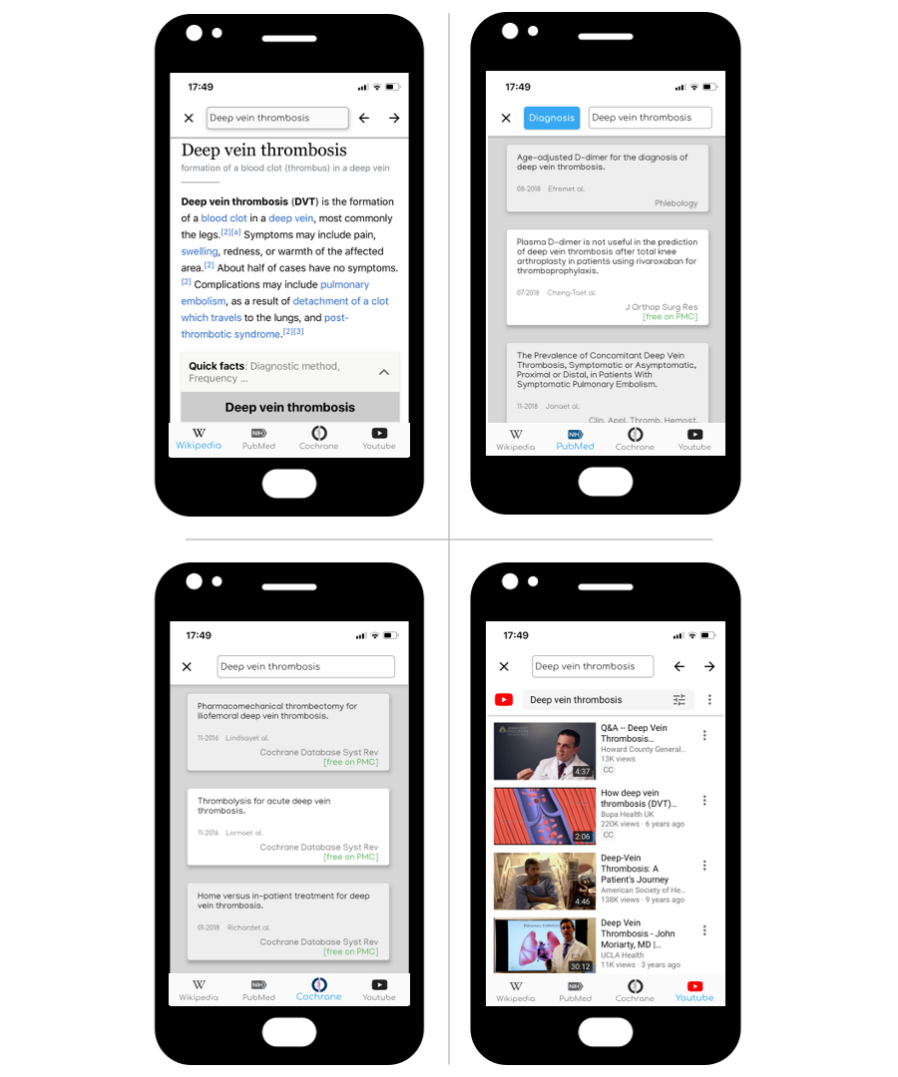
Search results for “Deep Vein Thrombosis” on the federated search platform. At the time of the study, the platform queried Wikipedia (top, left), PubMed (“diagnosis” category; top, right), the Cochrane library (bottom, left), and YouTube (bottom, right).

Federated searching describes a system that implements a single query concurrently across multiple disparate collections of information. The federated search engine used in this study is a free experimental platform called *SciScanner* that centralizes popular health-information resources [34]. At the time of the study, searches were implemented across a series of authoritative (PubMed and the Cochrane library), nonauthoritative (YouTube), and community‐built (Wikipedia) resources in tandem. These were chosen based on prior research identifying the most commonly used, free resources among health care professionals [35-39] and because of the ease with which a simple search string could be used and adapted for each data source while maximizing the consistency of these results [40]. For example, the “Clinical Queries” search strategies were implemented in the system to aid users in finding different types of content on PubMed (eg, related to diagnosis, clinical prediction, or treatment best practices) [41]. The results for a single search query could be accessed for each resource on the app home screen (Figure 1).

Following recruitment and before completion of the protocol, participants were required to download and register an account with the SciScanner federated search platform. This authentication process facilitated the tracking of participants’ account-linked information search and retrieval behaviors. The timestamps of all search events, the accounts associated with these events, the search queries themselves, and the resources used for each search query were logged by the system and could be exported in a comma-separated values (CSV) file format for further statistical analysis.

##### Web Logs

Web logs were acquired using a web browser extension. This browser extension was installed on the computer provided to participants during the completion of their examination. When opened, the browser loaded a blank homepage; participants were allowed to select their preferred sites and search engines. The browser extension captured a similar collection of metrics as those described previously, which were used to quantify information search and retrieval behaviors: search queries, associated timestamps, and the URLs of the webpages that were accessed were logged by the extension. These logs were manually exported in a CSV file format following completion of each examination for further analysis.

### Data Preparation

All web and federated search log files were accrued in a single database after participants had completed the test protocol for each “assisted” condition and were filtered to remove setup (eg, log-in events to institutional networks) and duplicate events. All log files were then grouped into sessions based on the timestamps of the first and last log, which were cross-referenced with the recorded date, start time, and duration of the examination. Each session was then manually segmented by the primary author into individual information-seeking events based on search queries and the timestamps of every event; consecutive search queries that were deemed to be thematically related (eg, “sensation to medial tibia,” “nerve supply to medial leg,” and “saphenous nerve”) and those that occurred within a similar timeframe were grouped as a single information-seeking event. Information-seeking events were then partitioned into 3 periods: primary, intermediate, and terminal. The primary period related to the first search that was logged by participants in an information-seeking session or the first search logged in a thematically similar series of consecutive search segments. Terminal periods were defined as the last search logged by participants in an information-seeking session or the last search logged in a thematically similar series of consecutive search segments. Intermediate periods included consecutive logs between the primary and terminal periods.

**Figure 2:**
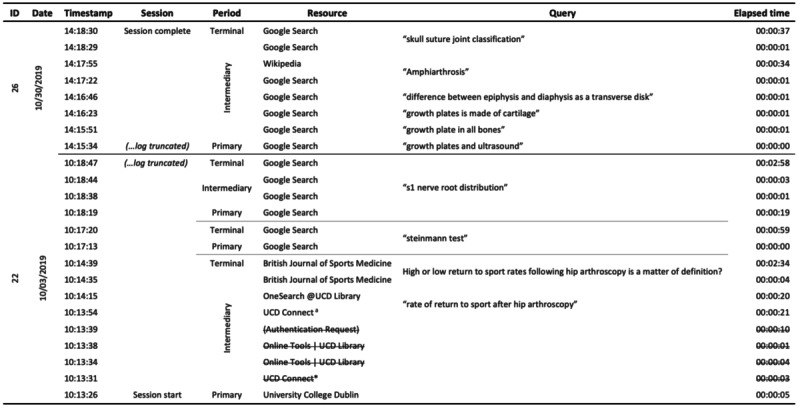
An example collection of search sessions segmented into primary, intermediate, and terminal search periods. Strikethrough text depicts that events would have been removed during data preparation, as they coincided with log-in and authentication to an institutional website.

For each period of an information-seeking event, the resources that were accessed were identified. This was automatically recorded in the federated search logs by design and was determined based on the website URL for the web logs. The number of hits and the accumulated time for each hit were then determined for each resource for the entire test cohort. Segmentation, partitioning, and preparation for data analysis are displayed in Figure 2.

### Sample Size

Previous research on knowledge acquisition using web-based resources among medical trainees identified a 15% difference between assisted and nonassisted assessment scores (Cohen *d*=0.86) [21,26]. On this basis, a sample of 18 pairs of physiotherapists was deemed sufficient to achieve experimental power. To account for a 5% (2 of 36) potential dropout, 38 participants (19 pairs) were recruited; however, there were no dropouts, so all participants were included in the analysis.

### Outcomes

We adopted a 3-tiered analysis paradigm for our outcomes. The first tier was related to the MCQ results (examination-level analysis), the second to the individual questions of the MCQ (question-level analysis), and the third was related to the log files recorded in instances where there was an information need (log-level analysis; assisted conditions only).

#### Examination-Level Analysis

The salient outcomes for the examination-level analysis included participants’ overall MCQ scores and their examination times for each condition. In addition, the total number of information needs was a salient outcome for examinations completed in the “assisted” conditions. Total examination scores were computed without implementing any negative marking.

#### Question-Level Analysis

The salient outcomes for the question-level analysis included the presence or absence of an information need for each question, the time spent answering each question, and whether the question was answered correctly or incorrectly.

#### Log-Level Analysis

The salient outcomes for the log-level analysis included the number of hits, the time spent per hit, and the digital information resource that was used.

### Statistical Analysis

The demographics of the participating sample were represented using descriptive statistics.

#### Examination-Level Analysis

Generalized estimating equations (GEEs) were used to assess differences in examination scores (assisted with a web browser, assisted with a federated search engine, and unassisted conditions) and information needs (assisted with a web browser and assisted with federated search engine conditions) with examination time included as a covariate using an exchangeable correlation structure. The model was corrected for dependent observations by including the participants’ identifying code as a subject effect. The *a priori p* value for this analysis was set at *P*<.05.

#### Question-Level Analysis

A separate model GEE was defined to evaluate the effect of different conditions on individual answers in the presence and absence of an information need with question time included as a covariate in the model. The model was corrected for dependent observations by including the participants’ identifying code as a subject effect. The *a priori p* value for this analysis was set at *P*<.05.

#### Log-Level Analysis

Log data for all web-based and federated search–based information resources were represented using means with SDs or medians with IQR, where appropriate, for each period of the information trail. GEEs were used to assess the differences in the time spent per “hit” in each of the 3 periods of the information trail (primary, intermediate, and tertiary) for each of the assisted conditions using an exchangeable correlation structure. This model was corrected for dependent observations by including the participants’ identifying code as a subject effect. The *a priori p* value for this analysis was set at *P*<.05.

All data analyses were performed using Statistical Package for the Social Sciences version 26 (IBM Corp) and Microsoft Excel.

## Results

### Participant Characteristics

**Figure 3:**
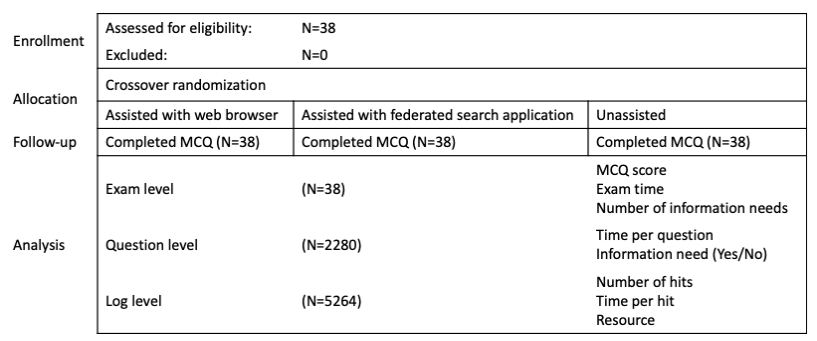
CONSORT (Consolidated Standards of Reporting Trials) diagram of the study design. Note that information needs were considered in the assisted conditions only. MCQ: multiple-choice questionnaire.

A total of 38 physiotherapists fully participated in the study, completing an examination under each of the 3 experimental conditions in random order using Vickers’ block randomization [42]. The demographic characteristics of the included sample are presented in Table 1. All tests were conducted in the 6-month period from June 2019 to January 2020. All participants completed all 3 conditions within a 1-month (4 week) period of enrolling in the study. A CONSORT diagram is presented in Figure 3 [43].

**Table 1:** Characteristics of participants.

Characteristics	Values
Male, n	19
Female, n	19
Age (years), mean (95% CI)	28.6 (27.5-29.7)
Number of years practicing, mean (95% CI)	5.4 (4.4-6.3)
Number of individual patient encounters per week, mean (95% CI)	26.1 (22.5-29.7)

### Examination-Level Analysis

The examination-level GEE estimated a main effect for condition (*P*<.001). Examination scores differed significantly between the unassisted condition (mean examination score 47.11%, SD 12.7) and both assisted conditions (mean federated search condition 57.5%, SD 12.8, *P*=.001; web search condition 63.3%, SD 12%, *P*=.02). The mean examination score and examination duration for the 3 experimental conditions, and the mean number of information needs for the assisted conditions are presented in Table 2.

**Table 2:** Results of the examination-level analysis for the 3 experimental conditions including the mean examination score, duration, and number of information needs for the assisted conditions (with 95% CI).

Exam parameter		Number of information needs, mean (95% CI)	Time (min), mean (95% CI)	Score (%), mean (95% CI)
**Assisted**				
	Federated search	11 (10-12)	34 (29-39)	58 (53-62)
	Web search	11 (10-12)	27 (23-31)	63 (59-67)
Unassisted		N/A^a^	12 (11-14)	47 (43-51)

^a^N/A: not applicable.

### Question-Level Analysis

Participants experienced 845 information needs out of a total of 1520 questions, with a rate of 55.6% (assisted conditions only). The question-level GEE was used to further investigate the relationship between individual information needs and answers (correct and incorrect). This GEE estimated a main effect for condition (*P*<.001) and the presence of an information need (*P*=.009). An analysis of the parameter estimates associated with these main effects revealed that participants were more likely to answer correctly in both the federated search condition (*P*=.003) and the web browser condition (*P*<.001) compared with the unassisted condition.

Despite the observation of a main effect for the presence of an information need, there were no significant differences based on the parameter estimates at the level of *P*<.05 in the rate of answering individual questions correctly in the presence or absence of an information need (*P*=.06).

The average rate of answering correctly for each condition in the absence or presence of an information need is presented in Table 3.

**Table 3:** Mean rate of answering correctly (with 95% CI) for each condition in the absence or presence of an information need.

Condition	Rate of answering correctly (%), mean (95% CI)		
	Federated search condition	Web search condition	Unassisted condition
Yes	62.1 (57.4-66.7)	65.2 (60.6-69.7)	N/A^a^
No	72.5 (67.6-77.2)	65.1 (60-70.2)	43.7 (40.1-47.2)

^a^N/A: not applicable.

### Log Analysis

The 845 information needs corresponded to 1987 web logs and 3277 app logs. This data set of app and web logs comprised all participants’ information-seeking sessions, which themselves included multiple information-seeking events (or information trails) split into primary, intermediate, and terminal periods.

Table 4 presents the most popular resources used during the primary, intermediate, and terminal periods in the web and app log conditions.

In the web-assisted condition, participants spent a total of 00:45:25 min (383 hits) in the primary period, 04:14:55 hours in the intermediate period (1356 hits), and 06:26:43 hours in the terminal period (248 hits). This corresponded with a median time per resource of 1 second in the primary period, 4 seconds per resource in the intermediate period, and 86 seconds per resource in the terminal period.

In the federated search condition, participants spent a total of 01:47:21 hours (486 hits) in the primary period, 08:42:24 hours in the intermediate period (2076 hits), and 04:24:06 hours in the terminal period (668 hits). This corresponded with a median time per resource of 3 seconds in the primary period, 4 seconds per resource in the intermediate period, and 5 seconds per resource in the terminal period.

The log GEE estimated a main effect for condition and for the period of the information trail (*P*<.001). There was also a significant main effect for the interaction between condition and period (*P*<.001). An analysis of the parameter estimates associated with these main effects revealed that participants spent less time per hit in the primary and intermediate periods for the web search condition compared with the federated search condition, and more time in the terminal period in the web search condition than in the federated search condition. The median and mean values for each condition stratified by search period are presented in Table 5.

**Table 4:** Most popular resources used during the primary, intermediate, and terminal periods for the web and federated search conditions.

Period, search condition, and resource				Hits, n		Cumulative (%)		Time (h:min:s)		Cumulative (%)
**Primary**										
	**Web browser**									
		Google Search	359		94		00:39:55		88	
		PubMed-NCBI^a^	9		96		00:02:35		94	
		Physio-pedia	9		98		00:01:32		97	
		ResearchGate	1		99		00:00:54		99	
		Google Scholar	2		99		00:00:15		99	
		Ovid	2		100		00:00:09		100	
	**Federated search**									
		Wikipedia	304		62		00:53:35		50	
		PubMed	131		88		00:36:32		84	
		YouTube	36		95		00:12:13		95	
		Cochrane	23		100		00:05:03		100	
**Intermediate**										
	**Web browser**									
		Google Search	858		63		01:33:50		37	
		PubMed-NCBI	141		74		00:49:23		56	
		Wikipedia	57		78		00:19:38		64	
		Physio-pedia	48		81		00:18:57		71	
		Google Scholar	47		85		00:10:07		75	
		British Journal of Sports Medicine	22		87		00:01:13		76	
		ResearchGate	14		88		00:04:25		77	
		Mayo Clinic	9		88		00:03:22		79	
		Sci-hub	8		89		00:00:56		79	
		Cochrane library	8		89		00:01:54		80	
	**Federated search**									
		PubMed	937		46		03:56:48		46	
		Wikipedia	848		87		03:20:38		85	
		Cochrane	133		94		00:25:31		90	
		YouTube	129		100		00:51:18		100	
**Terminal**										
	**Web browser**									
		Google Search	56		23		01:33:49		24	
		PubMed-NCBI	45		41		01:20:12		45	
		Physio-pedia	33		54		00:53:53		59	
		Wikipedia	26		65		00:35:35		68	
		British Journal of Sports Medicine	12		69		00:18:05		73	
		Mayo clinic	5		71		00:05:17		74	
		ResearchGate	5		73		00:01:27		75	
		Teachmeanatomy	4		75		00:06:19		76	
		Google Scholar	3		76		00:03:04		77	
		Mananatomy	3		77		00:05:16		78	
	**Federated search**									
		Wikipedia	366		55		01:47:48		42	
		PubMed	205		85		01:22:54		75	
		YouTube	57		94		00:50:49		94	
		Cochrane	42		100		00:14:04		100	

^a^NCBI: National Center for Biotechnology Information.

**Table 5:** Median and mean values of time spent per resource for the web and federated condition stratified by search period.

Search period		Primary (second)	Intermediate (second)	Tertiary (second)
**Federated search**				
	Median (IQR)	3 (11)	4 (12)	5 (26)
	Mean (95% CI)	13.3 (10.6-15.9)	15.1 (13.6-16.6)	24 (20.3-27.2)
**Web**				
	Median (IQR)	1 (7)	4 (14)	86 (55)
	Mean (95% CI)	7.1 (5.3-9)	11.3 (10.4-12.2)	169.8 (40-299.5)

## Discussion

### Principal Findings

#### Frequency of Information Needs

In this study, we sought to confirm a series of hypotheses. The first concerned the frequency with which physiotherapists experience information needs. Information needs were specifically defined: they were induced during a theoretical examination and anchored to participants’ access or nonaccess of digital information resources in the 2 “assisted” experimental conditions. Under this definition, participants experienced an information need in 55.59% (845/1520) of theoretical examination questions. In clinical environments, previous research has shown that medical physicians report experiencing an information need with varying frequencies. In the study by Covell et al [5], medical physicians had an information need in 67% of their patient encounters, whereas Sackett and Straus [44] observed 98 questions during the care of 166 hospitalized patients in a 30-day period (a rate of 59%). Among family care physicians, Ely et al [45] observed information needs at a rate of 3.2 (or 32%) for every 10 patient visits, while, more recently, Izcovich et al [46] documented 1.2 questions per patient encounter. It is important to note that by anchoring the existence of a need to information-seeking behavior, we could not evaluate *information needs* in the unassisted condition.

Regardless of how information needs are defined, timely translation of research evidence to the care pathway is a policy priority of many health research systems [47], yet many obstacles block the channels by which such translation occurs [48]. Ultimately, these obstacles impede effective evidence translation [49].

#### The Use of Web-Based Resources to Fulfill Information Needs

Barriers to obtaining information include time [40,50], accessibility [40,50,51], and limited personal skills [40], yet web-connected digital technologies could overcome these barriers and enable clinicians to fulfill their information needs at the point of care [1]. This contributed to our second confirmatory hypothesis that digital information resources accessed via the web could be used to fulfill information needs, thus improving the overall MCQ examination score. In agreement with this hypothesis, participants exhibited a mean improvement of 10% (for the federated search condition) and 16% (for the web condition) in the overall examination score compared with the unassisted condition. Our analysis at the resolution of individual questions further revealed that participants were more likely to answer correctly in either of the assisted conditions but that there were no differences in the rate of answering correctly in the presence or absence of an information need. Specifically, participants answered 63.7% (538/845) of questions correctly in the presence of an information need, compared with 68.7% (464/675) of questions in the absence of an information need. There was no statistically significant difference between these rates, suggesting that the availability of assistance improved participants’ likelihood of answering a question correctly at a similar rate to that when they were confident of knowing the answer (and therefore did not seek assistance, even if it was available) or to a level where they were sufficiently confident to guess despite the expectation that they would be marked negatively for doing so.

Previous researchers have sought to affect knowledge acquisition as assessed via MCQ examinations among health care professionals with seminars [52], tutorials [53], and course modules [54]. Course materials are generally developed using the frameworks of evidence-based medicine [55,56]. However, this does not reflect real-world practice, as health care professionals [57,58] autonomously use search engines and nonauthoritative, community‐built content to fulfill their information needs, prioritizing efficiency, familiarity, accessibility, and organization with “just the right” volume, variety, and scope to fulfill their needs [40]. The apparent dissonance between the types of resources that academic staff and researchers encourage health care professionals to use [55,56], those they report using [59,60], and those they actually use [61] informed our hypothesis-generating research questions. Through the log file analysis, we evaluated the digital information resources used by our cohort of physiotherapists in an unconfined web environment and those accessed via a federated search engine that was developed for the purposes of this study.

Reflecting the available body of observational research evaluating the browsing behaviors of the general population for health-related information [62,63], we observed a preference mainly for search engines and nonauthoritative, community‐built content at all stages of the information-seeking journey. Specifically, in the web-assisted condition, Google was the most popular resource used at the start of the information-seeking journey (359/383, 93.7% of total hits; 00:39:55 of 00:45:25, 88% of the total time). The variety of resources then increased in the “intermediate” period, yet Google was still the most popular (858/1351, 63.51% of total hits; 01:33:50 of 04:13:03, 37% of total time), followed by PubMed (141/1351, 10.44% of total hits; 00:49:23 of 04:13:03, 20% of total time) and Wikipedia (57/1351, 4.22% of total hits; 00:19:38 of 04:13:03, 8% of total time). The terminal period contained the last resources used by participants before their submission of an answer in the MCQ. Therefore, these resources were assumed to be principally responsible for fulfilling participants’ information needs, an assumption reflected in the relative amount of time spent in this period (median duration of 86 seconds), compared with the primary (median duration of 1 second) and intermediate (median duration of 4 seconds) periods. In the terminal period, Google searches again accounted for the largest number of hits with 22.5% (56/248) of the total, which corresponded to 01:33:49, or 25% of the total duration (06:18:13), followed by PubMed (45/248, 18.1% of total hits; 01:20:12 of 06:18:13, 21% of total duration), Physio-pedia (33/248, 13.3% of total hits; 00:53:53 of 06:18:13, 14% of total duration), and Wikipedia (26/248, 10.4% of total hits; 00:35:35 of 06:18:13, 9% of total duration). That Google, PubMed, and Wikipedia were the most popular resources among a cohort of physiotherapists is in agreement with a small body of prior research in other health care professional groups [61]. However, that Google in particular is heavily relied upon as both a directory to other resources and as a resource itself is potentially problematic, owing to its propensity to display results in a “filter bubble” [64]. Indeed, Google was the only search engine used by participants in this study, and its tendency to display previews of the information contained in individual websites potentially bypasses the need to visit these websites and to properly apprise the evidence [65]. The impact of this in the context of health care delivery has yet to be formally evaluated, but the available body of research suggests that the use of Google may not always be conducive to acquiring valid and reliable health-related information [66,67].

The federated search engine in this study was designed as an alternative mode of assistance, bypassing the need for traditional web search filter bubbles and improving the efficiency of navigating specific academic databases. Unsurprisingly, due to the constraints on the resources that were included in the federated search, a less diverse array of information resources were observed in the federated search condition. Specifically, Wikipedia accounted for the greatest number of hits in the primary period (304/494, 61.5% of hits; 00:53:35 of 01:47:23, 50% of time), PubMed accounted for the greatest number of hits in the intermediate period (937 of 2047 hits and 03:56:48 of 08:34:14, or 45.77% of both total hits and time), and Wikipedia accounted for the greatest number of hits in the terminal period (366/670, 54.6% of total hits; 01:47:48 of 04:15:35, 42% time), suggesting that participants sought out these resources despite the differences in their mode of delivery. Although the difference was not statistically significant, participants spent longer doing the examination in the federated search condition (34 min vs 27 min in the web condition). However, participants did spend significantly less time in the terminal period (a median duration of 5 seconds, compared with 86 seconds in the web condition) and clicked on a greater number of resources (there were 1290, or 65% more hits in the federated search condition compared with the web condition). In summary, these findings may suggest that the federated search was less effective in finding relevant and useful information, required more effort to locate that information, or that participants used the primary, intermediate, and terminal resources together to fulfill their information needs, rather than reaching an information *end-point* through a terminal resource as in the web condition. In relation to our second hypothesis-generating research question, that there was no significant difference in the examination scores between the 2 assisted conditions, and no significant difference in the rate with which questions were answered correctly or incorrectly in the presence or absence of an information need, would suggest the latter conclusions to be more likely.

### Limitations

Despite the methodological strengths of the crossover experimental design and the insights garnered via the use of web log analysis to determine what digital information resources were used to fulfill information needs and the novelty of evaluating these outcomes among physiotherapists, this study is not without limitations. First, an MCQ was used as a surrogate stimulus for information needs and may not be an accurate representation of the clinical setting. The validity of the MCQ questions as a measure of physiotherapy knowledge and by extension, health care delivery, was assumed on the basis that they were from the examinations of recognized accreditation bodies. It must be acknowledged that although these questions are used to assess physiotherapists’ capacity to practice, they may not actually represent the information needs of the authentic clinical encounter. Second, this study does not address an enduring question as to whether the fulfillment of clinical information needs alters treatment practices and patients’ outcomes; it has always been assumed, but never proven [46], that the translation of the latest high-quality research evidence to the care pathway optimizes clinical practice behaviors and improves patients’ outcomes. Finally, although the use of a federated search portal system provides a useful comparator for unconstrained web browsing in evidence search and retrieval patterns and their effect on MCQ examination scores, the results presented in this study for this portal system (ie, SciScanner) are unique to it and have limited external applicability for other portal systems.

### Conclusions

On the basis of an MCQ examination protocol, we identified that physiotherapists experienced an information need in 55.59% (845/1520) of theoretical questions and, when they were provided with access to digital information resources accessed via the web or a federated search software app, the fulfillment of these needs was associated with improved rates of answering examination questions correctly. The physiotherapists in this study exhibited a high preference for Google as both a directory and a resource, with Wikipedia and PubMed being the next most popular resources. The implications of relying heavily on Google as a search and retrieval mechanism for health-related information warrants further investigation, whereas the emulation of these findings in an authentic clinical setting would be an important research pursuit in the future.

## Abbreviations

CONSORT-EHEALTH: Consolidated Standards of Reporting Trials of Electronic and Mobile Health Applications and Online Telehealth
CSV: comma-separated values
GEE: generalized estimating equation
MCQ: multiple-choice questionnaire
NPTE: National Physical Therapy Exam
PCE: Physiotherapy Competency Exam
